# Diarrhoea deaths and disability-adjusted life years attributable to suboptimal breastfeeding practices in Nigeria: findings from the global burden of disease study 2016

**DOI:** 10.1186/s13006-019-0198-9

**Published:** 2019-01-09

**Authors:** Felix Akpojene Ogbo, Anselm Okoro, Bolajoko O. Olusanya, Jacob Olusanya, Ifegwu K. Ifegwu, Akorede O. Awosemo, Pascal Ogeleka, Andrew Page

**Affiliations:** 1 Translational Health Research Institute, School of Medicine, Western Sydney University, Penrith, NSW Australia; 2 Prescot Specialist Medical Centre, Welfare Quarters, Makurdi, Benue State Nigeria; 3 Independent Public Health Consultant, 4 Joy Street Cooperative City Gardens, Sabon Lugbe, Abuja, Nigeria; 4 Centre for Healthy Start Initiative, 286A Corporation Drive, Dolphin Estate, Ikoyi, Lagos, Nigeria

**Keywords:** Breastfeeding, Diarrhoea, Infant, Children, Mortality, Nigeria

## Abstract

**Background:**

In Nigeria, diarrhoea contributes significantly to childhood morbidity and mortality, with suboptimal breastfeeding practices playing a key role. The present study aimed to report on diarrhoea deaths and disability-adjusted life years (DALYs) among children aged under five years attributable to suboptimal breastfeeding practices in Nigeria.

**Methods:**

This study used data from the Global Burden of Disease study 2016, which estimated mortality from diarrhoea in the Cause of Death Ensemble model. Suboptimal breastfeeding was assessed as a combination of non-exclusive breastfeeding and discontinued breastfeeding. The comparative risk assessment approach was used to estimate the attributable burden of diarrhoea deaths and DALYs due to suboptimal breastfeeding practices in the spatial-temporal Gaussian Process Regression tool.

**Results:**

In 2016, suboptimal breastfeeding practices accounted for an estimated 56.5% (95% uncertainty intervals [UI]: 47.5, 68.3) of diarrhoea deaths in the late neonatal period, 39.0% (31.0, 46.3) in post-neonatal period, 39.0% (31.3, 46.20) in infancy period and 22.8% (16.9, 29.9) in children aged under five years in Nigeria. In the same year, 22,371 (14,259, 32,746) total diarrhoea deaths in children under five years could be attributed to suboptimal breastfeeding practices. DALYs from diarrhoea attributable to suboptimal breastfeeding practices was 1.9 million (1.2, 2.8 million) among children under five years in 2016. Between 1990 and 2016, the proportion of children who died from diarrhoea due to suboptimal breastfeeding did not change substantially across all age groups in Nigeria.

**Conclusions:**

Suboptimal breastfeeding practices remain a significant contributor to diarrhoea mortality and disability among children under five years in Nigeria. The study builds on previously published works on breastfeeding practices in Nigeria and provides evidence to support calls for the scale-up of efforts to improve infant feeding outcomes and reduce diarrhoea burden in Nigeria.

**Electronic supplementary material:**

The online version of this article (10.1186/s13006-019-0198-9) contains supplementary material, which is available to authorized users.

## Background

Globally, there is strong evidence for the protective effect of exclusive breastfeeding (EBF) on childhood diseases such as diarrhoea, a significant contributor to under-5 mortality in many developing countries, including Nigeria [1, 2]. Exclusive breastfeeding has also been shown to reduce the risk of childhood respiratory tract infection and overweight/obesity, and improved cognitive functioning of children [1]. Exclusive breastfeeding is not only beneficial to the child but also to the mother, the family and the community. For the breastfeeding mother and the family, EBF enhances family planning, reduces the risk of breast and ovarian cancers [1], and increases household and workplace productivity due to the low cost of breast milk and reduced child sick days [3]. Based on the advantages of appropriate breastfeeding practices, the World Health Organisation and United Nations Children’s Funds (WHO/UNICEF) recommend breastfeeding initiation for all newborns within the first hour of birth, EBF for the first six months of life and continued breastfeeding until two years and beyond [4].

Following the WHO/UNICEF recommendation, many countries (e.g., Ghana and Sri Lanka [5]) have implemented a range of initiatives to improve childhood nutrition, with subsequent improvements in breastfeeding outcomes. In Nigeria, however, previous studies have reported that the prevalence of EBF has remained well below expected level, an average of 16% between 1999 and 2013 [6–9], compared to countries such as Tanzania (63%) [9, 10], Uganda (63%) [9] and Ghana (54%) [11]. Recent studies from Nigeria have suggested that optimal breastfeeding is protective against diarrhoeal diseases among children younger than five years of age [2, 12]. However, since the 1970s, diarrhoea has remained a leading cause of deaths among children under five years in Nigeria [13–15], and the country has the second highest number of under-five mortality from diarrhoea worldwide, after India [14].

The most appropriate public health interventions are evidence-based, cost-effective and culturally-appropriate, and targeted at improving human health and reducing harm to individuals from disease [16]. To improve child survival and health outcomes, as well as ensure efficient use of limited health resources in Nigeria, it is essential to understand not only the distribution of suboptimal breastfeeding but also how current inappropriate breastfeeding practices impact health outcomes among children under five years. Using the comparative risk assessment (CRA) approach as described by Murray and Lopez [17], the burden of diarrhoea morbidity and mortality attributable to suboptimal breastfeeding can be estimated to provide an in-depth understanding on the impact of suboptimal breastfeeding on diarrhoea among children aged under five years at the population level in Nigeria to inform policy decision-making processes.

The Global Burden of Disease, Injury and Risk Factor (GBD) study provides comprehensive and comparable health data for 195 countries and territories to facilitate timely policy decision-making for many health focus areas, including the assessment of disease burden attributable to suboptimal breastfeeding. The GBD study uses high-level scientific methodology to generate comprehensive results, and the interpretation of those findings may be complex for policy-makers and/or public health researchers who are not trained in GBD methods and results. Additionally, the huge size of the results makes them impossible to be discussed in detail in the annual GBD capstone publications [13, 15, 18, 19]. This has led to an additional description and detailed discussion of the results for various health focus areas and locations globally [20–25]. Therefore, distilling the robust GBD information in the context of the burden of diarrhoea attributable to suboptimal breastfeeding practices in Nigeria is essential for national public health researchers and policy decision-makers and international donors to inform targeted breastfeeding programmes.

To the authors’ knowledge, this is the first country-level study to report on under-5 deaths and disability-adjusted life years (DALYs) from diarrhoea that could be attributed to suboptimal breastfeeding practices in Nigeria, as the country remains Africa’s largest contributor to under-5 mortality rate [13]. DALYs are the number of years lost due to ill-health, disability or premature death [19]. The present study aimed to describe diarrhoea deaths and DALYs among children under five years attributable to suboptimal breastfeeding practices in Nigeria using data from GBD 2016.

## Methods

### Study setting

Nigeria is located on the West Africa coast and occupies approximately 923,768 km^2^ of land extending from the fringes of the Sahara Desert in the north to the Gulf of Guinea on the Atlantic coast in the south. Constitutionally, Nigeria is divided into 36 states and the Federal Capital Territory, Abuja. Each state is subdivided into local government areas (LGAs), and each LGA is divided into wards [26]. Nigeria is the most populous country in sub-Saharan Africa, with an estimated 186 million people (including more than 40 million children) in 2017 [27] and is one of the most diverse countries globally, with over 250 ethnic groups [28–30]. The Nigerian healthcare system is complex and comprises a public sector (at all levels of government) and private sector (i.e. private for-profit providers, not-for-profit providers, religious and traditional providers, and community-based providers) [26, 31, 32]. Despite the annual budgetary allocation for health by the Nigeria Federal Ministry of Health, the country is the largest recipient of developmental assistance for health in Africa [33].

### Data source

The present study was based on results from the GBD 2016 study that were specific to the Nigerian context for the years 1990, 1995, 2000, 2005, 2010 and 2016. The GBD study is a systematic and scientific effort that provides comparable estimates of incidence, prevalence, cause of morbidity and mortality, and health loss by age–sex–year–location and over time, for diseases, injuries and risk factors. The GBD 2016 complied with the Guidelines for Accurate and Transparent Health Estimates Reporting (GATHER) protocol, a global agreement that promotes best practice and transparency in reporting health estimates [34]. For this study, the overall data sources, evidence for risk-outcome pairs, the conceptual framework, estimation strategy and statistical analysis for the calculation of diarrhoea deaths and DALYs among children under five years attributable to suboptimal breastfeeding practices have been described elsewhere [14, 15, 18]. A list of data sources used for the estimation of diarrhoea deaths and DALYs due to suboptimal breastfeeding in Nigeria is provided in Additional file 1. Data relating to the published results and codes used for the GBD 2016 are available elsewhere [35, 36], respectively.

### Risk factors

Suboptimal breastfeeding practices were assessed as a combination of non-exclusive breastfeeding (non-EBF) and discontinued breastfeeding. Non-EBF was defined as the proportion of children younger than six months of age who received breast milk as the predominant source of nourishment (but which also received water and water-based drinks, such as fruit juice, ritual fluids, oral rehydration solution, syrups or drops of vitamins) or no breastfeeding. Discontinued breastfeeding was defined as the proportion of children aged 6–23 months who did not receive any breast milk [18]. In the study, the selection of suboptimal breastfeeding–diarrhoea association was based on the following criteria: (i) the importance of a risk factor to disease burden or policy based on previous work; (ii) availability of sufficient data and methods to allow estimation of exposure distributions in Nigeria; (iii) sufficient evidence for the association based on high-quality epidemiological studies; and (iv) evidence to support generalisability of effect sizes to populations [18, 37–39].

### Outcome variables

Mortality and DALYs from diarrhoea among children under five years were the main outcome variables. Diarrhoea mortality was categorised into late neonatal (7–27 days), post-neonatal (28 days–1 year), infant (0–12 months), child (1–4 year) and under-5 (0–59 months) [13]. DALYs are a summary metric of disease burden and are used for resource allocation and health priority setting [40], and are defined as the number of years lost due to ill-health, disability or premature death [19]. In the Nigeria context, the estimation of DALYs from diarrhoea attributable to suboptimal breastfeeding will assist policy decision-makers and health managers to effectively allocate the limited infant and young child feeding resources.

### Overview of analytical strategy

Diarrhoea mortality was modelled in the Cause of Death Ensemble model (CODEm), a Bayesian, hierarchical, ensemble modelling tool that used data from verbal autopsy studies in Nigeria [14, 15]. CODEm is a term used to describe the technique employed to combine various individual models (e.g., mixed-effects models and spatiotemporal Gaussian process regression) to investigate relative predictive power of each approach for cause of death, while accounting for various covariates (e.g., childhood undernutrition and safe water and sanitation) [15, 41]. This modelling tool was developed for the GBD study to estimate mortality due to diseases or injuries by sex, age, location and over time and has been shown to provide more accurate uncertainty estimates compared to other combination modelling strategy [15, 42, 43]. A detailed description of CODEm and its use in the estimation of diarrhoea mortality are provided elsewhere [41, 43].

The CRA framework [17] was used to estimate the attributable burden of diarrhoea due to suboptimal breastfeeding practices. The CRA approach evaluates how much of the burden of disease observed in a given year can be attributed to past exposure to a risk (suboptimal breastfeeding practices). The attributable burden is the reduction in the current disease burden that would have been possible if past exposure had been shifted to an alternative or counterfactual distribution of risk exposure. Murray and Lopez [17] classified the counterfactual exposure distribution into theoretical, plausible, feasible and cost-effective minimum risk. The focus of this study is on the theoretical minimum risk level (TMREL), which is the level of risk exposure that minimises risk at the population level. For the TMREL in this study, all children younger than six months were exclusively breastfed, while all children received breastmilk until two years of age [18].

In the estimation of the attributable burden of suboptimal breastfeeding practices, four components were included: number of deaths, years of life lost (YLLs), years lived with disability (YLDs) or DALYs; the exposure levels for suboptimal breastfeeding; the relative risk of diarrhoea due to suboptimal breastfeeding; and the counterfactual level of suboptimal breastfeeding. The calculation of attributable DALYs for suboptimal breastfeeding and diarrhoea were equal to DALYs for diarrhoea multiplied by the population attributable fraction (PAF) for suboptimal breastfeeding and diarrhoea for a given age group–sex–year and country. A similar approach was applied for the calculation of attributable mortality, YLLs or YLDs [18, 37–39].

For the suboptimal breastfeeding–diarrhoea association, the attributable burden was estimated using the following equation in GBD 2016 [18]:

$$ {AB}_{jasct}=\sum \limits_{o=1}^w{DALY}_{oasct}{PAF}_{joasct} $$

Where *AB*_*jasct*_ is the attributable burden for risk factor *j* in age group *a*, sex *s*, country *c* and year *t*. *DALY*_*oasct*_ is DALYs for cause *o* (of *w* relevant outcomes for risk factor *j*) in age group *a*, sex *s*, country *c* and year *t*. *PAF*_*joasct*_ is the population attributable fraction (PAF) for cause *o* due to risk factor *j* in age group *a*, sex *s*, country *c* and year *t*. Attributable deaths, YLLs and YLDs were computed by substituting these metrics for DALYs in the equation [18].

The *PAF*_*joasct*_ for suboptimal breastfeeding was estimated using the following equation: [18].

$$ {PAF}_{joas ct}=\frac{\sum_{\mathrm{x}=1}^{\mathrm{u}}{RR}_{joas t}\left(\mathrm{x}\right){P}_{jas ct}\left(\mathrm{x}\right)\hbox{-} {RR}_{joas}\left({TMRE}_{jas}\right)}{\sum_{\mathrm{x}=1}^{\mathrm{u}}{RR}_{joas t}\left(\mathrm{x}\right){P}_{jas ct}\left(\mathrm{x}\right)} $$

*RR*_*joas*_(*x*) is the relative risk as a function of exposure level *x* for the risk factor (suboptimal breastfeeding) *j*, cause *o*, age-group *a* and sex *s*. *l* is the lowest level of exposure, and *u* is the highest level of exposure observed. *P*_*jasct*_(*x*) is the distribution of exposure for risk *j* in age-group *a*, sex *s*, country *c* and year *t*. *TMREL*_*jas*_ is the theoretical minimum risk exposure level for risk factor *j*, age group *a* and sex *s*. The equations highlight the four key components by cause, age, sex, country and year that go into estimating the burden attributable to a risk factor: the number of deaths, YLLs, YLDs or DALYs; exposure levels for a risk factor; relative risk of a given outcome due to exposure; and the counterfactual level of risk factor exposure [18]. In this study, the risk factor (*j*) denotes suboptimal breastfeeding practices, while country (*c*) represents Nigeria.

The analyses were performed in the spatial-temporal Gaussian Process Regression tool, which adjusted for country-level covariates (i.e., lag distributed income per capita, maternal education and total fertility), incorporated the variance of the epidemiological data and model predictions [18]. The effect size (and corresponding 95% uncertainty intervals, UIs) for suboptimal breastfeeding was estimated for age group, sex, exposure level, mean exposure, the distribution of exposure across individuals, TMREL and burden rates. UIs are a range of values that are likely to include the correct estimate of health loss for a given cause. The estimation of UIs not only adjusts for sampling error but also captures uncertainty from several analytical modelling stages and adjusts for the type and quality of data sources [19, 25, 44]. A detailed description of the analytical strategy is provided in the 2016 GBD capstone publication [18].

## Results

### Diarrhoea deaths due to suboptimal breastfeeding practices

**Fig. 1:**
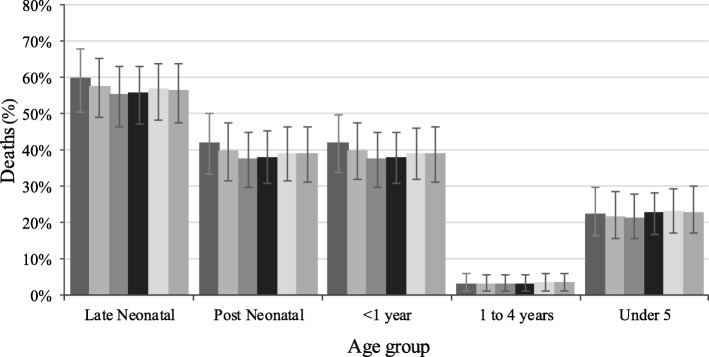
Percentage of diarrhoea deaths due to suboptimal breastfeeding practices in Nigeria, 1990–2016, . Error bars indicate 95% uncertainty intervals

In 2016, suboptimal breastfeeding practices accounted for 56.5% (95% uncertainty intervals [UI]: 47.5, 68.3) of diarrhoea deaths in the late neonatal period, 39.0% (31.0, 46.3) in the post-neonatal period, 39.0% (31.3, 46.20) in infancy and 22.8% (16.9, 29.9) in children younger than five years in Nigeria (Fig. 1). Regarding diarrhoea mortality numbers, the study showed that diarrhoea deaths were 22,371 (14,259, 32,746) among children under five years and 1550 (477, 3109) among children aged 1–4 years in 2016, possibly reflecting the age classification (Table 1). From 1990 to 2016, suboptimal breastfeeding practices accounted for a high proportion of diarrhoea deaths across all age groups, except for children aged 12–59 months, with an average of 3.0% (Fig. 1). Non-EBF accounted for higher proportions (between 20.7 and 57.7%) of suboptimal breastfeeding practices that could be attributed to diarrhoeal diseases in 2016 across all age groups compared to discontinued breastfeeding which varied from 1.3 to 3.5%, data not shown.

**Table 1:** Diarrhoea deaths due to suboptimal breastfeeding practices by age groups in Nigeria, 1990–2016

	1990	1995	2000	2005	2010	2016
Age	N (95%LUI, UUI)	N (95%LUI, UUI)	N (95%LUI, UUI)	N (95%LUI, UUI)	N (95%LUI, UUI)	N (95%LUI, UUI)
Late Neonatal	3328 (1983, 5163)	3346 (1835, 5485)	4160 (2396, 6705)	5542 (3255, 8512)	4026 (2359, 6206)	2337 (1267–3745)
Post Neonatal	14,301 (8031, 22,420)	13,810 (7628, 21,347)	18,159 (10,335, 27,877)	28,461 (17,592, 41,900)	27,283 (17,410, 39,021)	18,484 (11,334, 28,170)
< 1 year	17,629 (10,897, 26,199)	17,155 (10,255, 25,434)	22,319 (13,616, 33,173)	34,003 (22,288, 48,226)	31,309 (21,028, 44,218)	20,820 (13,032, 30,667)
1 to 4 years	1412 (459, 2851)	1333 (394, 2700)	1618 (496, 3271)	2315 (736, 4477)	2230 (767, 4479)	1550 (477, 3109)
Under 5	19,041 (12,086, 28,022)	18,488 (11,372, 27,561)	23,937 (14,822, 35,079)	36,318 (24,475, 50,617)	33,539 (22,533, 46,553)	22,371 (14,259, 32,746)

*N* number of deaths, *LUI* lower uncertainty interval, *UUI* upper uncertainty interval

### Diarrhoea burden due to suboptimal breastfeeding practices

**Fig. 2:**
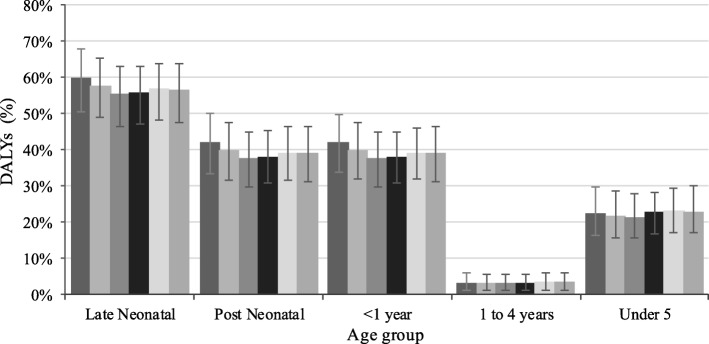
Percentage of DALYs from diarrhoea due to suboptimal breastfeeding practices by age in Nigeria, 1990–2016, . Error bars indicate 95% uncertainty intervals

In 2016, the burden of diarrhoea due to suboptimal breastfeeding practices was 1.9 million DALYs (1.2, 2.8 million) among children under five years, 1.8 million DALYs (1.1, 2.6 million) among infants and 1.6 million DALYs (984,294, 2.6 million) among post-neonates in Nigeria (Table 2). Proportionally, the burden of diarrhoea due to suboptimal breastfeeding was 56.5% (47.5, 68.3%) in the late neonatal period and 3% (1, 6%) in children aged 1–4 years in the same year (Fig. 2). Similarly, the numbers of fatal (YLLs) and non-fatal (YLDs) health outcomes that could be attributed to suboptimal breastfeeding practices were high among children under five years, infants and post-neonates (Tables 3 and 4).

**Table 2:** Disability-adjusted life years from diarrhoea due to suboptimal breastfeeding practices by age groups in Nigeria, 1990–2016

Age	1990	1995	2000	2005	2010	2016
	N (95%LUI, UUI)	N (95%LUI, UUI)	N (95%LUI, UUI)	N (95%LUI, UUI)	N (95%LUI, UUI)	N (95%LUI, UUI)
Late Neonatal	288,820 (172,129, 447,519)	290,406 (159,497, 475,681)	360,951 (208,204, 581,303)	480,731 (282,597, 737,800)	349,633 (205,147, 538,486)	203,243 (110,684, 325,252)
Post Neonatal	1,239,186 (698,375, 1,941,009)	1,197,696 (663,838, 1,845,742)	1,573,166 (898,786, 2,413,027)	2,461,430 (1,524,153, 3,618,053)	2,361,579 (1,511,544, 3,376,592)	1,602,738 (984,293, 2,439,842)
< 1 year	1,528,006 (946,701, 2,268,654)	1,488,102 (890,692, 2,202,046)	1,934,117 (1,183,464, 2,869,073)	2,942,161 (1,936,174, 4,170,749)	2,711,212 (1,825,806, 3,819,803)	1,805,981 (1,130,945, 2,655,800)
1 to 4 years	120,944 (39,237, 243,427)	114,487 (34,010, 232,016)	138,895 (42,980, 280,600)	197,957 (63,062, 381,471)	191,111 (65,743, 381,916)	134,135 (41,897, 267,318)
Under 5	1,648,950 (1,049,382, 2,421,292)	1,602,589 (990,134, 2,382,968	2,073,012 (1,283,023, 3,031,432)	3,140,117 (2,118,652, 4,376,115)	2,902,323 (1,955,679, 4,022,568)	1,940,116 (1,236,503, 2,831,861)

*N* number of disability-adjusted life years, *LUI* lower uncertainty interval, *UUI* upper uncertainty interval

**Table 3:** Years of life lost from diarrhoea due to suboptimal breastfeeding practices by age groups in Nigeria, 1990–2016

Age	1990	1995	2000	2005	2010	2016
	N (95%LUI, UUI)	N (95%LUI, UUI)	N (95%LUI, UUI)	N (95%LUI, UUI)	N (95%LUI, UUI)	N (95%LUI, UUI)
Late Neonatal	288,099 (171,612, 446,917)	289,628 (158,813, 474,789)	360,070 (207,435, 580,446)	479,725 (281,747, 736,857)	348,511 (204,183, 537,211)	202,259 (109,714, 324,182)
Post Neonatal	1,231,854 (691,740, 1,931,223)	1,189,524 (657,105, 1,838,754)	1,564,100 (890,207, 2,401,087)	2,451,459 (1,515,226, 3,608,950)	2,349,988 (1,499,597, 3,360,990)	1,591,898 (976,110, 2,426,160)
< 1 year	1,519,954 (939,862, 2,258,408)	1,479,152 (884,438, 2,193,353)	1,924,169 (1,173,651, 2,859,803)	2,931,185 (1,921,226, 4,155,885)	2,698,499 (1,812,384, 3,810,216)	1,794,157 (1,123,374, 2,642,472)
1 to 4 years	119,233 (38,742, 240,694)	112,547 (33,235, 227,982)	136,603 (41,900, 276,187)	195,470 (62,105, 378,025)	188,279 (64,739, 378,176)	130,910 (40,281, 262,508)
Under 5	1,639,186 (1,040,304, 2,411,586)	1,591,699 (979,752, 2,370,962)	2,060,772 (1,275,904, 3,021,501)	3,126,655 (2,107,270, 4,357,763)	2,886,777 (1,939,484, 4,007,743)	1,925,067 (1,225,301, 2,817,491)

*N* years of life lost, *LUI* lower uncertainty interval, *UUI* upper uncertainty interval

**Table 4:** Years of life lived with disability from diarrhoea due to suboptimal breastfeeding practices by age groups in Nigeria, 1990–2016

Age	1990	1995	2000	2005	2010	2016
	N (95%LUI, UUI)	N (95%LUI, UUI)	N (95%LUI, UUI)	N (95%LUI, UUI)	N (95%LUI, UUI)	N (95%LUI, UUI)
Late Neonatal	720 (1054, 461)	778 (1128, 500)	881 (1275, 554)	1006 (1467, 642)	1122 (1635, 720)	984 (1452, 633)
Post Neonatal	7332 (10,801, 4476)	8172 (11,996, 5098)	9066 (13,304, 5637)	9970 (14,653, 6217)	11,591 (16,867, 7215)	10,840 (15,834, 6851)
< 1 year	8053 (11,820, 4996)	8950 (12,999, 5614)	9948 (14,534, 6194)	10,976 (16,117, 6930)	12,713 (18,429, 7962)	11,824 (17,066, 7518)
1 to 4 years	1711 (3343, 554)	1940 (3702, 592)	2293 (4438, 689)	2486 (4748, 748)	2832 (5349, 891)	3225 (6273, 1022)
Under 5	9764 (14,296, 6063)	10,890 (16,154, 6804)	12,240 (17,946, 7518)	13,462 (19,650, 8474)	15,545 (23,029, 9699)	15,049 (22,374, 9356)

*N* years of life lived with disability, *LUI* lower uncertainty interval, *UUI* upper uncertainty interval

## Discussion

The current study showed that more than half (56.5%) of diarrhoea deaths in the late-neonatal period could be attributed to suboptimal breastfeeding practices in Nigeria. From 1990 to 2016, the proportion of children who died from diarrhoea due to suboptimal breastfeeding practices did not change substantially across all age groups in Nigeria. The increased burden of diarrhoea deaths and health loss due to suboptimal breastfeeding practices among children aged under five years reflect the age classification.

A reduction or modification to a risk factor exposure is an essential strategy for improving population health, and the quantification of the attributable burden of risk factors in relation to their specific outcomes could provide a roadmap for the effectiveness of public health efforts [18, 24]. The evidence of suboptimal breastfeeding practices as a risk factor of diarrhoeal morbidity and mortality is well-documented in the scientific literature [1, 45]. Using this evidence and the CRA approach, the present study indicated that more than half of the diarrhoea deaths in the late neonatal period could be attributed to suboptimal breastfeeding practices in Nigeria. Although the Government of Nigeria had introduced many national and subnational programs to improve breastfeeding practices, previous nationwide studies from Nigeria have shown that the proportion of mothers who engage in suboptimal breastfeeding practices have remained high since the year 1990 [7, 8, 46]. The lack of substantial improvement in infant feeding practices calls for a comprehensive and measurable strategy to improve child nutrition in Nigeria.

Globally, Nigeria has the second highest under-5 mortality, behind India, and diarrhoeal disease is a significant contributor to this disease burden [14, 15]. The current study has shown that the improvement in breastfeeding practices would be helpful to increase child survival and extend the longevity of life. More recently, the United Nations General Assembly declared the decade (2016–2025) as the “Decade of Action on Nutrition”, with the aim of galvanising global, political and developmental effort on nutrition at the country level [47]. While this resolution is essential for improving nutrition (including breastfeeding) and to consolidate other nutrition programmes in Nigeria, the country would require strong political commitment and increases in healthcare funding to improve breastfeeding practices. These measures are vital in Nigeria because of inadequate implementation or a lack of sustainability of previous global, regional and national healthcare agendas in the country [7, 32, 48].

The present study indicated that there was a substantial health loss (in terms of DALYs) from diarrhoea due to suboptimal breastfeeding among children under five years in Nigeria. Many studies conducted in Nigeria have shown that most mothers have good knowledge and a positive attitude towards breastfeeding [26, 49–51]. However, the uptake of optimal breastfeeding practices such as EBF for infants under six months has been limited since 1990 [8, 52]. Risk factors for suboptimal breastfeeding in Nigerian children have been described in more detail elsewhere [8, 32, 46, 52]. These include a lack of maternal education, poor household wealth, limited access to health services, maternal employment, socio-cultural beliefs relating to breastfeeding, commercial promotion of infant formula, limited political will and poor healthcare funding for maternal and child health programmes. This study provides a current evidence base to inform more sustained effort and advocacy for optimal breastfeeding, and to engage stakeholders and policy decision-makers at all levels of government in the implementation or review of breastfeeding initiatives to improve child health outcomes.

These findings have locally-relevant policy implications for both national and subnational governments in Nigeria and international donor agencies aiming to improve child nutrition in the next decade in the country. Previous nationally representative studies conducted in Nigeria have suggested broader evidence-based interventions and policy responses to improve breastfeeding with subsequent impact on diarrhoea mortality and DALYs in Nigerian children [7, 9, 12, 32, 53]. Key aspects of those initiatives could be categorised as government, facility-based or community-based measures. At the government-level, these include a stronger political commitment at all levels and increased funding, better accountability in healthcare spending, enforcement of relevant legislation and a refinement of current global infant and young child feeding agendas to high-priority populations in Nigeria [32].

At the facility level, the training of healthcare professionals and traditional birth attendants in the provision of appropriate nutrition information to mothers and strengthening of the baby friendly hospital initiative are strategies to improve breastfeeding in Nigeria [32]. At the community level, the involvement of family members in health information sessions relating to infant feeding (a baby-friendly community initiative) that are tailored to the specific socio-cultural context in which women raise their children, would also increase child survival in Nigeria [32]. Furthermore, those government, facility-based or community-based initiatives should focus on all mothers, especially those from low socio-economic status groups, mothers who give birth at home, and primiparous mothers to improve EBF, particularly in the neonatal period, with impact on diarrhoeal disease burden in Nigeria. Studies that evaluate strategies to improve infant feeding practices, current policy initiatives and funding allocations, as well as strengthening data collection points at the subnational level are needed in Nigeria.

The study has a number of limitations, which have been described in detail in previous GBD publications [18, 19, 37–39]. Briefly, the attributable burden of disease formula (eq. 1) multiplies PAFs by deaths, YLLs, YLDs, or DALYs. Firstly, the limitations associated with the calculation of mortality, YLLs, YLDs, and DALYs applied to this study [19]. For instance, in the estimation of diarrhoea mortality, there were differences in the case definitions and reporting approaches, as well as a lack of vital registration and other high-quality data at the national and subnational levels in Nigeria. The negative impact of ethnic diversity on infant feeding outcomes has been previously highlighted in Nigeria [26, 54, 55], suggesting that subnational data on infant feeding practices would be more robust and accurate in estimating their impact on diarrhoea. The improvement in the collection of high-quality epidemiologic data for diarrhoea by cause, age, sex, location and management in Nigeria would help to provide targeted and more effective interventions in improving child survival in the country. Secondly, the GBD study used pooled relative risks from prospective observational studies for the association between suboptimal breastfeeding and diarrhoea [45] to calculate the PAFs, but well-conducted randomised control trials may indicate different relative risks. Thirdly, publication bias relating to the relative risks may also be another limitation. Fourth, the non-inclusion of other breastfeeding indicators such as timely initiation of breastfeeding may also be a limitation of the study given its impact on diarrhoea in developing communities [56, 57].

## Conclusions

Strategic policy initiatives to improve population health should be informed by comprehensive and scientific evidence on the causes of deaths and health loss arising from risk factor exposure. The present study aimed to describe the burden of diarrhoea deaths and DALYs among children aged under five years attributable to suboptimal breastfeeding practices in Nigeria between 1990 and 2016 based on GBD 2016 findings. The study indicated that suboptimal breastfeeding practices are a major contributor to diarrhoea-related deaths and disability among children under five years and build on previously published evidence on the impact of suboptimal breastfeeding practices on diarrhoea in Nigeria. It also provides evidence to support calls for the scale-up of efforts to improve infant feeding outcomes and subsequent reduction in diarrhoea burden in Nigeria. Initiatives such as strong political commitment at all levels of government and increased healthcare funding, targeted community- and facility-based initiatives are needed in Nigeria to improve child survival.

## Additional file

Additional file 1: Data sources used for estimation of diarrhoea deaths and DALYs due to suboptimal breastfeeding in Nigeria in the Global Burden of Diseases (GBD) study. (DOCX 13 kb)

## Abbreviations

CRA: Comparative risk assessment
DALYs: Disability-adjusted life years
EBF: Exclusive breastfeeding
GBD: Global burden of disease
UI: Uncertainty intervals
UNICEF: United Nations Children’s Funds
WHO: World Health organisation
YLDs: Years lived with disability
YLLs: Years of life lost
