# Supplementary material for: Diarrhoea deaths and disability-adjusted life years attributable to suboptimal breastfeeding practices in Nigeria: findings from the global burden of disease study 2016
Source: Int Breastfeed J. 2019 Jan 9;14:4. doi: 10.1186/s13006-019-0198-9 (PMC6327380; doi:10.1186/s13006-019-0198-9)
Supplement: Supplementary file 1 — Data sources used for estimation of diarrhoea deaths and DALYs due to suboptimal breastfeeding in Nigeria in the Global Burden of Diseases (GBD) study. (DOCX 13 kb) [file 13006_2019_198_MOESM1_ESM.docx]

**Supplementary Table 1: Data sources used for estimation of diarrhoea deaths and DALYs due to suboptimal breastfeeding in Nigeria in the Global Burden of Diseases (GBD) study** (Source: <http://ghdx.healthdata.org/gbd-2016/data-input-sources>)

| **Serial No** | **Citation** |
| --- | --- |
|  | Federal Ministry of Health (Nigeria), Macro Systems, Inc.; Institute for Resource Development, National Population Bureau (Nigeria). Nigeria - Ondo Special Demographic and Health Survey 1986-1987. Columbia, United States: Macro Systems, Inc. |
|  | Federal Office of Statistics (Nigeria), Macro International, Inc.; Institute for Resource Development. Nigeria Demographic and Health Survey 1990. Calverton, United States: Macro International, Inc. |
|  | National Population Commission of Nigeria, ORC Macro, UK Department for International Development (DFID), United Nations Children's Fund (UNICEF), United Nations Population Fund (UNFPA). Nigeria Demographic and Health Survey 2003. Calverton, United States: ORC Macro. |
|  | National Population Commission of Nigeria and Macro International, Inc. Nigeria Demographic and Health Survey 2008. Calverton, United States: Macro International, Inc, 2009. |
|  | Central Bank of Nigeria, National Bureau of Statistics (Nigeria), Nigerian Communications Commission (NCC). Nigeria General Household Survey 2007. Abuja, Nigeria: National Bureau of Statistics (Nigeria). |
|  | Central Bank of Nigeria, National Bureau of Statistics (Nigeria), Nigerian Communications Commission (NCC). Nigeria General Household Survey 2008. |
|  | National Bureau of Statistics (Nigeria), United Nations Children's Fund (UNICEF). Nigeria Multiple Indicator Cluster Survey 1999. Abuja, Nigeria: National Bureau of Statistics (Nigeria). |
|  | United Nations Children's Fund (UNICEF), National Bureau of Statistics (Nigeria). Nigeria Multiple Indicator Cluster Survey 2007. New York, United States: United Nations Children's Fund (UNICEF). |
|  | National Bureau of Statistics (Nigeria), United Nations Children's Fund (UNICEF). Nigeria Multiple Indicator Cluster Survey 2011. New York, United States: United Nations Children's Fund (UNICEF), 2013. |
